# Genome-Wide Transcriptomic Analysis of Intestinal Tissue to Assess the Impact of Nutrition and a Secondary Nematode Challenge in Lactating Rats

**DOI:** 10.1371/journal.pone.0020771

**Published:** 2011-06-16

**Authors:** Spiridoula Athanasiadou, Leigh A. Jones, Stewart T. G. Burgess, Ilias Kyriazakis, Alan D. Pemberton, Jos G. M. Houdijk, John F. Huntley

**Affiliations:** 1 Animal Health Group, Scottish Agricultural College, Bush Estate, Penicuik, United Kingdom; 2 Division of Parasitology, Moredun Research Institute, Pentlands Science Park, Bush Loan, Penicuik, United Kingdom; 3 Veterinary Faculty, University of Thessaly, Karditsa, Greece; 4 Royal (Dick) School of Veterinary Studies, The Roslin Institute, University of Edinburgh, Roslin, United Kingdom; Dana-Farber Cancer Institute, United States of America

## Abstract

**Background:**

Gastrointestinal nematode infection is a major challenge to the health and welfare of mammals. Although mammals eventually acquire immunity to nematodes, this breaks down around parturition, which renders periparturient mammals susceptible to re-infection and an infection source for their offspring. Nutrient supplementation reduces the extent of periparturient parasitism, but the underlying mechanisms remain unclear. Here, we use a genome wide approach to assess the effects of protein supplementation on gene expression in the small intestine of periparturient rats following nematode re-infection.

**Methodology/Principal Findings:**

The use of a rat whole genome expression microarray (Affymetrix Gene 1.0ST) showed significant differential regulation of 91 genes in the small intestine of lactating rats, re-infected with *Nippostrongylus brasiliensis* compared to controls; affected functions included immune cell trafficking, cell-mediated responses and antigen presentation. Genes with a previously described role in immune response to nematodes, such as mast cell proteases, and intelectin, and others newly associated with nematode expulsion, such as anterior gradient homolog 2 were identified. Protein supplementation resulted in significant differential regulation of 64 genes; affected functions included protein synthesis, cellular function and maintenance. It increased cell metabolism, evident from the high number of non-coding RNA and the increased synthesis of ribosomal proteins. It regulated immune responses, through T-cell activation and proliferation. The up-regulation of transcription factor forkhead box P1 in unsupplemented, parasitised hosts may be indicative of a delayed immune response in these animals.

**Conclusions/Significance:**

This study provides the first evidence for nutritional regulation of genes related to immunity to nematodes at the site of parasitism, during expulsion. Additionally it reveals genes induced following secondary parasite challenge in lactating mammals, not previously associated with parasite expulsion. This work is a first step towards defining disease predisposition, identifying markers for nutritional imbalance and developing sustainable measures for parasite control in domestic mammals.

## Introduction

Periparturient relaxation of immunity (PPRI) in mammals is a well described phenomenon, whereby an already established immunity to a historic infection weakens, allowing successful colonization by a pathogen during late pregnancy and early lactation. This has been reported in a number of parasitic infections across a number of mammalian species, including man [1]. For free living mammals, PPRI drives the epidemiology of the parasitic infection; periparturient dams become a potent source of infection for their parasite naive offspring. Studies on PPRI towards nematode parasites have repeatedly demonstrated that nutrient supplementation and protein supplementation in particular, can reduce its extent. Such beneficial effects of protein supplementation have been demonstrated in both rodent [2], [3] and livestock hosts [4], [5]. Lactating rats offered a high protein diet and administered a secondary challenge of the intestinal nematode *Nippostrongylus brasiliensis* showed a 70% reduction in worm burdens compared to their low protein fed counterparts [2]. High protein fed lactating rats also showed increased mucosal mast cells, eosinophils, and IgG2b, as well as circulating IgG [3], [6]. Mucosal mast cell numbers were negatively correlated with worm numbers in the small intestine of the rats [6].

Despite the accumulating evidence on the effects of dietary protein on the phenotype of parasitized hosts, the relationships between nutrition and immunity to nematodes at the molecular level are not known. Insights into such interactions are of major importance, to define disease predisposition and develop sustainable measures for parasite control in managed animals. One hypothesis is that the relationship between dietary protein and effector immune responses may be quantitative; immune responses and in particular antibody production, are known to be highly proteinaceous in nature, therefore it is anticipated that these will be sensitive to protein availability [7]. A second hypothesis, which does not preclude the first one, is that the relationship between dietary protein and immune responses to nematodes is qualitative; there is evidence that certain dietary nutrients can affect gene regulation, which in turn will affect endogenous protein synthesis at various levels. This is considered an important mechanism allowing hosts to adapt their physiological functions according to the supply of nutrients in the diet. For example, it has been shown that specific amino acids, such as leucine, can regulate gene expression during transcription, post-transcription, translation and post-translation [8], [9]. To date, there is no evidence on the consequences of dietary protein on expression of genes related to immunity to nematodes.

In the present study we aimed to explore the molecular interactions between nutrition and immunity to nematodes and investigate whether protein supplementation can affect transcriptional regulation of genes at the niche of the parasite infection, the small intestine, during nematode expulsion. Our hypothesis was that protein supplementation would regulate gene expression at transcript level, in particular those genes related to immunity to nematodes. To examine these interactions, we used a whole genome rat array to interrogate gene expression in the small intestine of periparturient rats, supplemented with dietary protein at time of nematode expulsion; we opted for the time point they experienced a reduction of 70% in nematode numbers, compared to their un-supplemented counterparts, which reflects a maximal phenotypic divergence [6], [10]. Our aim was achieved, as we have provided evidence for transcriptional regulation of genes related to immunity to nematodes, as a consequence of protein supplementation. We have also identified genes that have not been previously associated with nematode expulsion in mammals.

## Results

### Microarray analyses

#### Consequences of secondary parasite infection on gene expression

A total of 383 genes were significantly differentially expressed between lactating rats that were re-infected and the sham infected controls (irrespective of their nutritional protocol); of these 91 genes had greater than 1.5 fold change expression, either up- or down-regulated (Table S1). A small proportion of these (7 genes) were down-regulated following secondary infection, whereas the majority were up-regulated (Table 1). Amongst the top up-regulated transcripts following the secondary challenge were enzymes, such as mast cell proteases, dual oxidase maturation factor 2 (*DUOXA2*) and phospholipase A2, hormones, such as resistin like-β (*RETNLB*) and cytokine receptors, such as interleukin 1 receptor like-1 (*IL1R1*). An approximate 12- and 8-fold up-regulation of dual oxidase 2 (*DUOX2*) and *DUOXA2* respectively was observed between the rats that received a secondary parasite challenge and those that received the sham infection. Other genes identified here have not previously been associated with nematode expulsion in mammals, such as anterior gradient homologue 2 (*AGR2*). The 1.5-fold up-regulation of *AGR2* in the parasitized rats (Table S1) compared to controls was also validated by qPCR. The top down-regulated gene encoded the glucose-6-phosphatase catalytic subunit (*G6PC*), a catalytic enzyme associated with glucose homeostasis and neutrophil chemotaxis [11].

**Table 1:** Genes significantly differentially regulated by secondary challenge with *N.brasiliensis* in lactating rats (P<0.05, FC>2*, MTC 0.05).

Gene symbol	Gene description	Fold Change	Location	Type(s)
*DUOXA2*	Dual oxidase maturation factor 2	11.8	Cytoplasm	other
*DUOX2*	Dual oxidase 2	8.3	unknown	enzyme
*MCPT4*	Mast cell protease 4	7.7	Extracellular Space	peptidase
*PLA2G4C*	Phospholipase A2, group IVC (cytosolic, calcium-independent)	4.3	Cytoplasm	enzyme
*RETNLB*	Resistin like beta	3.9	Extracellular Space	other
*CD55*	CD55 molecule	3.7	Plasma Membrane	other
*TPSB2*	Tryptase beta 2	3.7	Extracellular Space	peptidase
*MCPT8*	Mast cell protease 8	3.5	Extracellular Space	peptidase
*TPSG1*	Tryptase gamma 1	3.2	Extracellular Space	peptidase
*IL1RL1*	Interleukin 1 receptor-like 1	3.1	Plasma Membrane	transmembrane receptor
*GSDMC*	Gasdermin C	3.0	Cytoplasm	other
*POSTN*	Periostin, osteoblast specific factor	3.0	Extracellular Space	other
*MCPT1*	Mast cell protease 1	2.9	Extracellular Space	peptidase
*CPA3*	Carboxypeptidase A3 (mast cell)	2.7	Extracellular Space	peptidase
*MCPT4*	Mast cell protease 4	2.6	Extracellular Space	peptidase
*GPX2*	Glutathione peroxidase 2 (gastrointestinal)	2.6	Cytoplasm	enzyme
*CNN1*	Calponin 1, basic, smooth muscle	2.6	Cytoplasm	other
*SRGN*	Serglycin	2.5	Extracellular Space	other
*DES*	Desmin	2.5	Cytoplasm	other
*SPINK4*	Serine peptidase inhibitor, Kazal type 4	2.5	Extracellular Space	other
*RGS13*	Regulator of G-protein signaling 13	2.5	Nucleus	other
*APOL3*	Apolipoprotein L, 3	2.4	Cytoplasm	transporter
*ACTG2*	Actin, gamma 2, smooth muscle, enteric	2.4	Cytoplasm	other
*ITLN1*	Intelectin 1 (galactofuranose binding)	2.3	Plasma Membrane	other
*ITGA5*	Integrin, alpha 5 (fibronectin receptor, alpha polypeptide)	2.2	Plasma Membrane	other
*SH2D6*	SH2 domain containing 6	2.2	unknown	other
*LGALS1*	Lectin, galactoside-binding, soluble, 1	2.2	Extracellular Space	other
*CMA1*	Chymase 1, mast cell	2.1	Extracellular Space	peptidase
*MMP10*	Matrix metallopeptidase 10 (stromelysin 2)	2.0	Extracellular Space	peptidase
*NKG7*	Natural killer cell group 7 sequence	2.0	Plasma Membrane	other
*DNASE1*	Deoxyribonuclease I	−2.0	Extracellular Space	enzyme
*G6PC*	Glucose-6-phosphatase, catalytic subunit	−3.2	Cytoplasm	phosphatase

*For extended list of genes (FC>1.5) see Table S1.

The biological processes and canonical pathways significantly affected by the secondary parasite infection in the lactating rat are seen in Tables 2 and 3. Gene ontology and pathway analysis identified the biological processes and pathways significantly overrepresented in the intestinal mucosa as a consequence of parasitic infection. The top gene ontology functions included cellular movement, immune cell trafficking, and cell-mediated immune response, whereas the top pathways affected included eicosanoid signalling, leukocyte extravasation signalling, and Integrin-linked kinase signalling (for P values see Tables 2 and 3).

**Table 2:** Top biological processes significantly affected by secondary challenge with *Nippostrongylus brasiliensis* in lactating rats.

Category	Enrichment (P-value)*	Molecule counts
Cellular Movement	3.94E-11-5E-03	33
Respiratory Disease	3.96E-11-5E-03	22
Haematological System Development and Function	2.54E-10-5E-03	25
Immune Cell Trafficking	2.54E-10-5E-03	23
Inflammatory Disease	1.26E-09-5E-03	31
Inflammatory Response	1.34E-09-5E-03	31
Genetic Disorder	2.83E-09-4.87E-03	56
Cell-To-Cell Signalling and Interaction	8.43E-09-5E-03	24
Tissue Development	6.69E-08-5E-03	20
Lipid Metabolism	1.33E-07-5E-03	17
Molecular Transport	1.33E-07-5E-03	27
Small Molecule Biochemistry	1.33E-07-5E-03	20
Cell Death	2.29E-07-5E-03	31
DNA Replication, Recombination, and Repair	2.52E-07-5E-03	13
Cardiovascular Disease	6.23E-07-5E-03	32
Organismal Functions	7.85E-07-7.37E-05	8
Tissue Morphology	8.94E-07-1.04E-03	13
Cell Morphology	1.65E-06-5E-03	16
Cellular Development	1.65E-06-5E-03	25
Cardiovascular System Development and Function	2.53E-06-5E-03	18
Organ Morphology	2.53E-06-1.47E-04	7
Cell-mediated Immune Response	2.84E-06-5E-03	8
Connective Tissue Disorders	3.05E-06-4.56E-03	20
Skeletal and Muscular Disorders	3.05E-06-4.56E-03	31
Skeletal and Muscular System Development and Function	3.28E-06-5E-03	16
Cellular Growth and Proliferation	1.02E-05-3.99E-03	30
Embryonic Development	1.07E-05-5E-03	8
Protein Degradation	1.11E-05-1.11E-05	8
Protein Synthesis	1.11E-05-1.98E-03	10
Cell Signaling	1.14E-05-1.2E-03	15

*The P value reported here is a range between the most and least significant result, for the various molecules represented in each biological process.

**Table 3:** Canonical pathways significantly affected by secondary challenge with *Nippostrongylus brasiliensis* in lactating rats.

Canonical pathways	−log(p-value)	Ratio	Molecules
Eicosanoid Signaling	4.78E00	6.02E-02	5
ILK Signaling	4.53E00	3.76E-02	7
Leukocyte Extravasation Signaling	3.49E00	3.09E-02	6
IL-17 Signaling	3.34E00	5.41E-02	4
Caveolar-mediated Endocytosis Signaling	3.29E00	4.82E-02	6
Actin Cytoskeleton Signaling	3.2E00	2.58E-02	5
Arachidonic Acid Metabolism	3.12E00	2.21E-02	4
TR/RXR Activation	3.05E00	4.12E-02	4
Virus Entry via Endocytic Pathways	3.05E00	4.17E-02	4
Atherosclerosis Signaling	2.82E00	3.57E-02	4
Glioma Invasiveness Signaling	2.61E00	5.26E-02	3
Agrin Interactions at Neuromuscular Junction	2.32E00	4.35E-02	3
PAK Signaling	2.04E00	2.94E-02	3
FXR/RXR Activation	2.01E00	2.91E-02	3
NRF2-mediated Oxidative Stress Response	1.98E00	2.19E-02	4
Oncostatin M Signaling	1.9E00	5.71E-02	2
Coagulation System	1.83E00	5.41E-02	2
MIF Regulation of Innate Immunity	1.83E00	4.35E-02	2
Rac Signaling	1.83E00	2.5E-02	3
RhoA Signaling	1.78E00	2.73E-02	3
CCR3 Signaling in Eosinophils	1.77E00	2.5E-02	3
Cdc42 Signaling	1.77E00	2.44E-02	3
Hepatic Fibrosis/Hepatic Stellate Cell Activation	1.58E00	2.24E-02	3
Phospholipase C Signaling	1.57E00	1.58E-02	4
Galactose Metabolism	1.51E00	1.74E-02	2
TREM1 Signaling	1.48E00	2.9E-02	2

**Figure 1:**
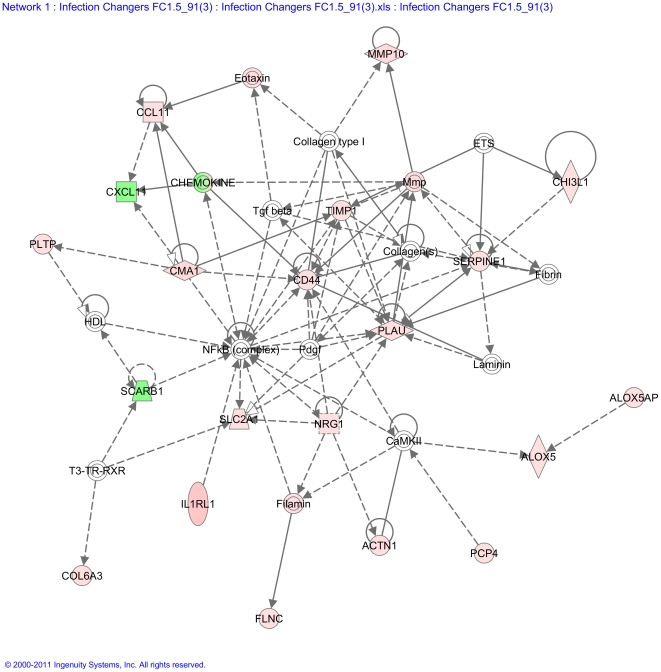
Graphical representation of network 1 as affected by secondary parasite challenge. Network score = 38. Individual nodes represent protein functions with relationships represented by edges. Nodes are colored by change in gene expression, red indicating up-regulation in infected rats, green indicating down-regulation in infected rats and white indicating that the gene/factor was not differentially expressed but showed a defined relationship to the other genes in the network. Arrows indicate directional relationships between genes.

In addition to gene function and pathway analysis, a further stage of analysis was performed, which allowed the identification of biological relationships that could otherwise be missed by conventional analysis. All differentially expressed genes were used for network analysis; the formation of networks links the various pathways and functions together, based on information derived from published studies to reveal further relationships between genes. Analysis identified a number of key networks; the one presented in Figure 1 had the highest score. This network is built around the transcriptional regulator nuclear factor kappa B (NF-kB) and the top functions overrepresented in this network include respiratory disease, cell-to-cell signalling and interaction and tissue development.

*In silico* transcription promoter analysis, which included all the differentially expressed genes, suggested that the transcription factor responsible for up-regulation of genes in secondary infected rats was likely SRF, with z score 14.69 and P = 1.814e-02. SRF stimulates cell proliferation/differentiation and is related to the mitogen-activated protein kinase (MAPK) pathway [12].

#### Consequences of protein nutrition on gene expression

Protein supplementation resulted in 70% reduction of worm counts in high protein (HP) compared to low protein (LP) rats [for detailed description of the phenotypes of these animals], [ see 6, 10]. From a total of 3500 genes that were significantly differentially expressed between rats given access to LP and HP (irrespective of their parasitic status), 65 transcripts had higher than 1.5 fold change expression (Table 4). From these, a small proportion (7 genes) was down-regulated in HP rats, whereas the majority were up-regulated. Amongst the top up-regulated transcripts in HP rats were enzymes, such as aldehyde dehydrogenase 1C (*ADH1C*) and solute carrier 11 A2 (*SLC11A2*), and interleukin receptors, such as *IL1R2*. From the down-regulated genes, the top regulated was *CD3G*, which is a T-cell surface glycoprotein [13].

**Table 4:** Genes significantly differentially regulated by protein supplementation in lactating rats (P<0.05, FC>1.5, MTC 0.05).

Gene symbol	Gene description	Fold Change	Location	Type(s)
*ADH1C*	Alcohol dehydrogenase 1C (class I), gamma polypeptide	3.6	Cytoplasm	enzyme
*SLC11A2*	Solute carrier family 11, member 2	2.0	Plasma Membrane	transporter
*ND5*	NADH dehydrogenase, subunit 5 (complex I)	1.9	Cytoplasm	enzyme
*RPS19*	Ribosomal protein S19	1.8	Cytoplasm	other
*RPS15A*	Ribosomal protein S15a	1.8	Cytoplasm	other
*FTL*	Ferritin, light polypeptide	1.7	Cytoplasm	other
*ATP5I*	ATP synthase, H+ transporting, subunit E	1.7	Cytoplasm	transporter
*IL1R2*	Interleukin 1 receptor, type II	1.7	Plasma Membrane	transmembrane receptor
*VSIG4*	V-set and immunoglobulin domain containing 4	1.7	Plasma Membrane	other
*HIST2H2AB*	Histone cluster 2, H2ab	1.7	Nucleus	other
*GM6744*	Predicted gene 6744	1.7	unknown	other
*RPL41*	Ribosomal protein L41	1.6	Cytoplasm	other
*CYBRD1*	Cytochrome b reductase 1	1.6	Cytoplasm	enzyme
*RPS12*	Ribosomal protein S12	1.6	Cytoplasm	other
*RPL41*	Ribosomal protein L41	1.6	Cytoplasm	other
*HIST1H2BK*	Histone cluster 1, H2bk	1.5	Nucleus	other
*RPS9*	Ribosomal protein S9	1.5	Cytoplasm	other
*HIST2H3C*	Histone cluster 2, H3c	1.5	Nucleus	other
*RPL22*	Ribosomal protein L22	1.5	Nucleus	other
*RGD1566344*	Similar to eukaryotic translation elongation factor 1 alpha 1	−1.5	unknown	other
*FAM190B*	Family with sequence similarity 190, member B	−1.5	unknown	other
*GUCA2A*	Guanylate cyclase activator 2A (guanylin)	−1.5	Extracellular Space	other
*PPAP2A*	Phosphatidic acid phosphatase type 2A	−1.5	Plasma Membrane	phosphatase
*SLC20A1*	Solute carrier family 20 (phosphate transporter), member 1	−1.6	Plasma Membrane	transporter
*PPARGC1A*	Peroxisome proliferator-activated receptor gamma, coactivator 1a	−1.6	Nucleus	transcription regulator
*VNN1*	Vanin 1	−1.6	Plasma Membrane	enzyme
*CD3G*	CD3g molecule, gamma (CD3-TCR complex)	−1.9	Plasma Membrane	transmembrane receptor
N/A	5S ribosomal RNA	6.1	Cytoplasm	non-coding RNA
N/A	novel pseudogene	1.7	Cytoplasm	non-coding RNA
N/A	U6 snRNA spliceosomal RNA	1.6	Nucleus	non-coding RNA
N/A	Small nucleolar RNA Z195/SNORD33	1.5	Nucleus	non-coding RNA
N/A	Y RNA	1.5	Nucleus	non-coding RNA
N/A	Mt tRNA	1.5	Cytoplasm	non-coding RNA
N/A	signal recognition particle (SRP) RNA	1.5	Cytoplasm	non-coding RNA

More than 20 of the transcripts up-regulated in the HP rats were for non-coding RNA, including ribosomal, spliceosomal and mitochondrial RNA (Table 4). These, in combination with up-regulation of transcripts associated with cell proliferation, such as ribosomal proteins L41, L41, S12 and S19 imply that HP rats showed increased metabolism and cell turnover compared to LP rats. In addition to these genes, other genes were identified, such as interleukin 1 receptor 2 (*IL1R2*) and V-set and immunoglobulin domain containing 4 (*VSIG4*) whose functions suggest that they may be of particular importance and their potential roles are discussed below.

**Figure 2:**
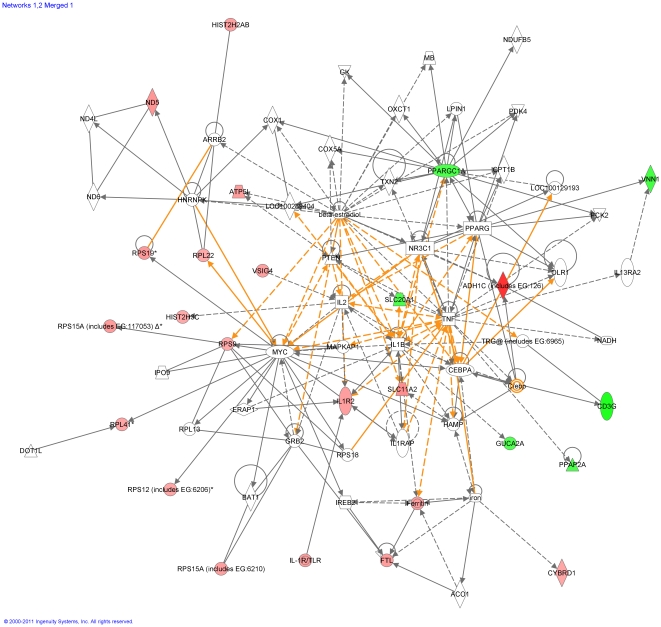
Graphical representation of merged networks 1 and 2 as affected by protein supplementation. Scores of individual networks: 33 and 19. Individual nodes represent protein functions with relationships represented by edges. Nodes are colored by change in gene expression, red indicating up-regulation in HP rats, green indicating down-regulation in HP rats and white indicating that the gene/factor was not differentially expressed but showed a defined relationship to the other genes in the network. Arrows indicate directional relationships between genes.

The biological processes and canonical pathways affected significantly by the level of dietary protein in the lactating rat are shown in Tables 5 and 6. The assignment of gene ontologies to each gene allowed them to be grouped and for the biological processes and pathways statistically overrepresented in the intestinal mucosa as a consequence of dietary protein intake to be identified. Top gene ontology functions included protein synthesis, cellular function and maintenance, whereas the top pathways affected included sphingolipid metabolism, peroxisome proliferator-activated receptors (*PPAR*) and p38 MAPK signalling (for P values see Tables 5 and 6). The first two pathways are associated with lipid metabolism, whereas the third one is a pro-inflammatory pathway. Network analyses showed the formation of two main networks from the molecules affected by the level of protein intake. These are merged and presented in Figure 2; the top functions overrepresented in these networks are protein synthesis, lipid metabolism, small molecule biochemistry and cellular function and maintenance. The two networks are formed around 3 major pro-inflammatory cytokines, IL1B, IL2, TNF and the implications of this will be discussed in the next section.

**Table 5:** Top biological processes significantly affected by protein supplementation in lactating rats.

Category	Enrichment(P value)*	Molecule counts
Protein Synthesis	1.13E-04-1.13E-04	5
Carbohydrate Metabolism	1.51E-03-4.21E-02	4
Cellular Function and Maintenance	1.51E-03-3.56E-02	3
Digestive System Development and Function	1.51E-03-1.51E-03	1
Genetic Disorder	1.51E-03-9E-03	3
Lipid Metabolism	1.51E-03-3.85E-02	4
Neurological Disease	1.51E-03-3.85E-02	2
Ophthalmic Disease	1.51E-03-1.51E-03	1
Skeletal and Muscular System Development and Function	1.51E-03-2.38E-02	2
Small Molecule Biochemistry	1.51E-03-4.28E-02	7
Tissue Morphology	1.51E-03-2.84E-02	5
Hematological Disease	1.63E-03-4.71E-02	6
Behavior	3.01E-03-1.2E-02	1
Cellular Development	3.01E-03-4.57E-02	5
Cellular Growth and Proliferation	3.01E-03-3.17E-02	4
Drug Metabolism	3.01E-03-2.68E-02	1
Gene Expression	3.01E-03-2.83E-02	1
Hematological System Development and Function	3.01E-03-4.57E-02	4
Hematopoiesis	3.01E-03-4.57E-02	2
Hepatic System Development and Function	3.01E-03-1.5E-02	1
Molecular Transport	3.01E-03-2.83E-02	5
Organismal Functions	3.01E-03-2.38E-02	1
Organismal Injury and Abnormalities	3.01E-03-3.85E-02	3
Vitamin and Mineral Metabolism	3.01E-03-3.41E-02	4
Cancer	4.51E-03-4.82E-02	7
Cell Death	4.51E-03-3.56E-02	2
Cell Morphology	4.51E-03-4.14E-02	2
Cellular Assembly and Organization	4.51E-03-3.99E-02	1
Nervous System Development and Function	4.51E-03-3.7E-02	4
Reproductive System Disease	4.51E-03-4.82E-02	2

*The P value reported here is a range between the most and least significant result, for the various molecules represented in each biological process.

**Table 6:** Canonical pathways significantly affected by protein supplementation in lactating rats.

Ingenuity Canonical Pathways	−log(p-value)	Ratio	Molecule counts
Sphingolipid Metabolism	2.14E00	1.79E-02	2
PPAR Signaling	2.13E00	2.04E-02	2
p38 MAPK Signaling	2.03E00	2.06E-02	2
Glycerolipid Metabolism	1.97E00	1.28E-02	2
Estrogen Receptor Signaling	1.93E00	1.68E-02	2
Lysine Biosynthesis	1.75E00	1.52E-02	1
Oxidative Phosphorylation	1.67E00	1.2E-02	2
PPARα/RXRα Activation	1.6E00	1.1E-02	2
Cytotoxic T Lymphocyte-mediated Apoptosis of Target Cells	1.4E00	3.12E-02	1

We analysed five different clusters of genes with similar expression profiles, as a consequence of the feeding treatment used. The analysis confirmed that biological functions over-presented in the clusters were cellular function and proliferation, with genes encoding ribosomal proteins representing 30% of a cluster (data not shown). The transcriptional promoter analysis demonstrated v-rel reticuloendotheliosis viral oncogene homolog A (RELA) as the most highly represented transcription factor binding site in the gene promoters analysed, with z score 7.524 and P = 4.033e-01 (non-significant scores).

#### Consequences of protein nutrition×secondary infection interactions

Seventy genes were found to be significantly differentially expressed in the interaction (no MTC was applied), with at least a 1.1 fold change, between rats given a secondary or a sham challenge and offered HP or LP (Table S2). From these, a proportion was shown to be differentially expressed in rats that received a secondary infection and offered HP compared to those offered LP; such differences were not present in the sham infected rats. A number of these genes were shown to be up-regulated in HP rats, such as *AGR2* and Fc fragment of IgG, low affinity IIIa, receptor (*FCGR3A*) with possible functions in innate and adaptive immunity [14], [15]. Other genes were down-regulated in infected HP rats compared to infected LP rats, such as *ZNF354A*, a transcription regulator [16]. In the absence of infection, genes such as *FCGR3A* and *NK2*, a transcription regulator [17], were also up-regulated in HP rats.

The biological processes and pathways statistically affected by the interactions between the parasitic challenge and protein supplementation are seen in Tables 7 and 8. The top gene ontology changes combine functions reported in the infection analysis, such as cell-to-cell signalling and inflammatory response, but also those that were affected by diet, such as amino acid metabolism and cellular function and maintenance. Three canonical pathways were found to be significantly affected by the interaction between infection and diet, which were cell cycle, TGF-β signalling and protein ubiquitination pathways (for P values see Tables 7 and 8). Network analysis identified the formation of two main networks, each contained 12 focus molecules. The functions overrepresented in the first include cell signalling and immunological disease, whereas the functions represented in the second include cell cycle and cell-to-cell signalling and interaction (data not shown).

**Table 7:** Top biological processes significantly affected by the interaction between protein supplementation and secondary challenge.

Category	Enrichment(P value)*	Molecule counts
Carbohydrate Metabolism	8E-04-4.87E-02	7
Cell-To-Cell Signaling and Interaction	1.1E-03-3.4E-02	9
Cellular Function and Maintenance	1.1E-03-3.77E-02	3
Inflammatory Response	1.1E-03-1.1E-03	2
Amino Acid Metabolism	3.83E-03-3.03E-02	1
Auditory and Vestibular System Development and Function	3.83E-03-3.83E-03	1
Cardiovascular System Development and Function	3.83E-03-4.87E-02	5
Cell Cycle	3.83E-03-3.77E-02	3
Cell Death	3.83E-03-1.52E-02	5
Cellular Assembly and Organization	3.83E-03-3.77E-02	4
Cellular Development	3.83E-03-3.77E-02	7
Cellular Growth and Proliferation	3.83E-03-4.87E-02	14
DNA Replication, Recombination, and Repair	3.83E-03-2.28E-02	2
Developmental Disorder	3.83E-03-4.87E-02	4
Embryonic Development	3.83E-03-4.87E-02	7
Gene Expression	3.83E-03-2.65E-02	12
Genetic Disorder	3.83E-03-4.87E-02	11
Hematological Disease	3.83E-03-3.4E-02	2
Lipid Metabolism	3.83E-03-4.14E-02	6
Molecular Transport	3.83E-03-4.9E-02	10
Nervous System Development and Function	3.83E-03-4.14E-02	3
Neurological Disease	3.83E-03-4.87E-02	8
Ophthalmic Disease	3.83E-03-3.4E-02	2
Organ Development	3.83E-03-4.51E-02	10
Organ Morphology	3.83E-03-3.4E-02	4
Psychological Disorders	3.83E-03-4.87E-02	2
Reproductive System Development and Function	3.83E-03-4.87E-02	5
Reproductive System Disease	3.83E-03-4.87E-02	4
Skeletal and Muscular System Development and Function	3.83E-03-4.14E-02	4
Small Molecule Biochemistry	3.83E-03-4.51E-02	10

*The P value reported here is a range between the most and least significant result, for the various molecules represented in each biological process.

**Table 8:** Pathways significantly affected by the interaction between protein supplementation and secondary challenge.

Ingenuity Canonical Pathways	−log(p-value)	Ratio	Molecule counts
Cell Cycle: G2/M DNA Damage Checkpoint Regulation	2.01E00	4.65E-02	2
TGF-β Signaling	1.45E00	2.41E-02	2
Protein Ubiquitination Pathway	1.45E00	1.49E-02	3

### Quantitative PCR verification

**Figure 3:**
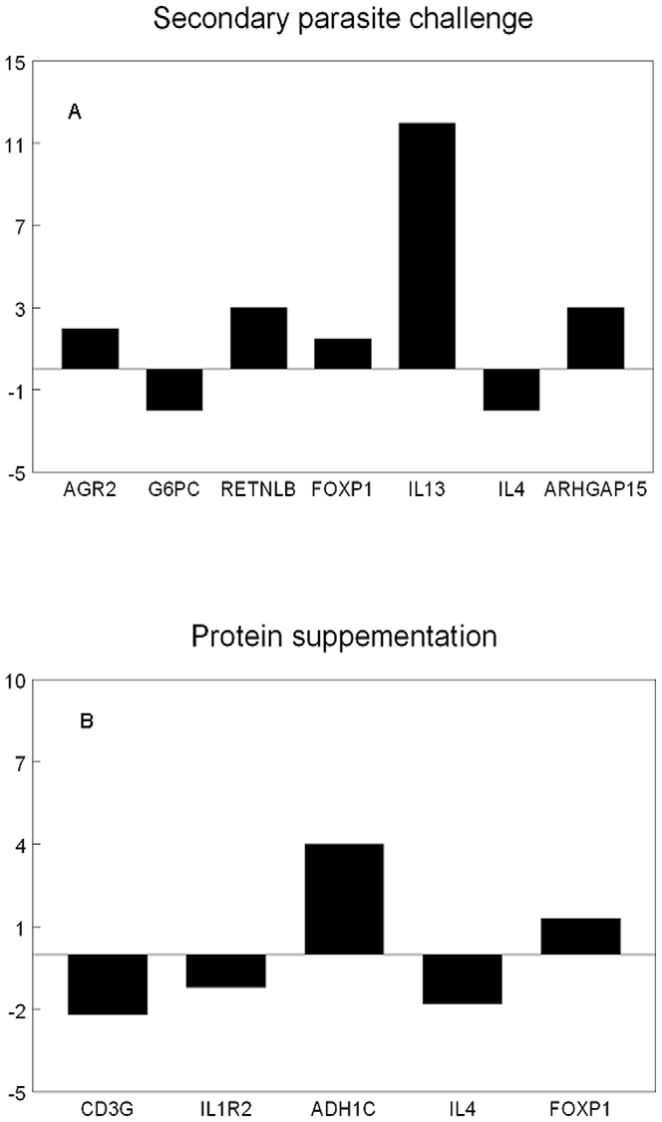
Confirmatory q RT PCR analysis of selected genes. Bars demonstrate fold change difference in mRNA of the genes shown in the x axes as a consequence of (a) secondary parasite challenge or (b) HP supplementation in comparison to non-infected or LP fed rats respectively.

To confirm the differential regulation pattern of genes observed by the microarray analysis, 11 genes were selected for confirmation analysis by quantitative RT PCR (Figure 3). Genes were selected either based on their gene expression values (one of the top 5 up- or down-regulated genes) such as *RETNLB*, *G6PC* and *ADH1C* or based on their biological relevance, such as *AGR2*, *CD3G*, *IL1R2*. *IL4* and *IL13* expression were also measured with qPCR as additional controls. These cytokines have known function as Th2 cytokines and their gene expression levels were expected to be increased following parasite infection. Gene concentrations were normalised using *β-actin* as our internal control gene, cloned and measured in the same way.

qPCR analysis demonstrated that for genes that were affected either by infection challenge (e.g. *AGR2*, *G6PC*, *RETNLB*) or diet (e.g. *IL1R2*, *CD3G*, *ADH1C*), gene expression levels of the microarray were verified (Figure 3). Fold change differences were the same in qPCR and the microarray and either reached statistical significance (P<0.05) or showed strong tendency (for *IL1R2* P = 0.065; for *CD3G* P = 0.06). The expression patterns of genes related to the interaction between protein nutrition and secondary infection, such as *GYG1* and *AGR2* were similar to those observed by the microarray. These expression values did not reach statistical significance (P>0.1), with the exception of *FOXP1*, which tended to be higher in LP infected rats (1.5-fold change up-regulation in LP infected rats; P = 0.07). qPCR analysis showed that *ARHGAP15* and *IL13* expression levels were significantly affected by the parasite challenge; similar expression patterns were observed in the microarray, although they failed to reach statistical significance. *IL4* had similar expression patterns in qPCR as in the microarray (P>0.1 between P and NP groups).

## Discussion

In the present study we utilized a well established lactating rodent –nematode parasite model to assess the nutritional sensitivity of host responses to gastrointestinal nematode parasitism, and employed a global approach to investigate gene expression patterns at the parasite niche, during the phase of their expulsion from the gastrointestinal tract. This model has been previously used to explore the effects of host nutrition on the level of parasitism and to define the phenotype of the parasitized host offered HP. In a series of animal studies, HP parasitized hosts showed consistently up to 70% lower parasite burden compared to those offered LP [2], [3], [6], [10], [18]. We have now shown for the first time that at least part of these nutritional effects on resistance to nematodes previously observed are generated at the transcript level in the intestinal mucosa. We have identified molecular links between protein supplementation and immunity that provide insights in the immune-regulatory effects of nutrition. The nutritional environment of the host affected a number of genes, pathways and networks related to nematode expulsion, irrespective of the presence of nematodes in the small intestine. Our results confirm a number of pathways already known to be affected by secondary parasite challenge and we reveal novel genes that have not previously been associated with *N. brasiliensis* secondary challenge.

Protective immunity and subsequent parasite expulsion during *N. brasiliensis* infection is driven by a Th2 response that is orchestrated by the secretion of cytokines such as IL-4, IL-5 and IL-13 and characterised by eosinophilia, mastocytosis, goblet cell hyperplasia and concurrent mucus and antibody production [19], [20]. This expulsion is known to be reliant on smooth muscle contraction and increased mucus production [21]. Here we provide evidence for regulation of genes and transcription factors related to both these mechanisms. Firstly, transcription promoter analysis demonstrated that *SRF* appears to be the primary transcription factor responsible for the orchestration of genes differentially expressed following secondary infection. *SRF* plays a central role in maintaining smooth muscle contraction [22] which is consistent with parasite expulsion. Up-regulation of genes such as *CNN1*, *TAGLN*, *CFL2* and *ACTG2* demonstrate smooth muscle cell hyperactivity at the time of sampling, which is likely to be mediated via integrin-linked kinase signalling (ILK signalling; see Table 3). There is also evidence for increased mucus production, with *ITLN1* and *AGR2* being significantly up-regulated. *ITLN* is a calcium dependent lectin, shown to be expressed by the Paneth cells in the small intestine of mouse [23] and the mucus neck cells in the abomasum of sheep [24]. It has been associated with mucus production, and high ITLN expression was induced during nematode expulsion in *Trichinella spiralis* and *N. brasiliensis* in rodents [25], [26] and *Teladorsagia circumcincta* infections in sheep [24], [27]. *AGR2* on the other hand has only recently been characterised as a protein disulfide isomerase, used by cells to aid protein folding and assembly [14]. It is expressed in intestinal epithelial cells and was found to be essential for the production of *MUC2*, a glycoprotein protective of the epithelium, also secreted by goblet cells [21]. Although the actual function of the gene is not known, it has been shown that mice lacking a functional copy of *AGR2* show increased susceptibility to intestinal infection [14]. To our knowledge, our study shows for the first time that *AGR2* is up-regulated during expulsion of *N. brasiliensis* in rats.

The two top regulated genes following the parasite challenge were those related to oxidation, *DUOXA2* and *DUOX2*; expression of these genes was up- regulated also in sheep and cattle during a gastrointestinal nematode infection [28], [29]. Other reactive oxidants have been shown to be up-regulated in rats infected with *N. brasiliensis*, and have been associated with parasite damage leading to subsequent expulsion [30]. In addition, it has recently been hypothesised that *DUOX* up-regulation may be related to protection of the microbiota in the intestine of blood feeding insects [31]. *DUOX*, together with a peroxidase, appear to form a dityrosine (oxidative product of tyrosine) network in the gut that decreases gut permeability to immune elicitors. These studies suggest that this network protects the microbiota in the intestine by preventing the activation of an epithelial cell induced immune response. Although not extensive, there is some evidence demonstrating interactions between parasites in the intestine and gut microbiota [32]; thus we would hypothesise that if intestinal nematodes have a negative impact on the microbial population in the gut, this may in turn stimulate host epithelial cells to trigger DUOX expression with the aim to exhibit a protective role towards the microbiota. This hypothesis remains to be tested.

The significant up-regulation of small non-coding RNA molecules following protein supplementation, irrespective of the infection status, such as Mt RNA and ribosomal RNA, indicates increased cell metabolism, turnover and consequently protein synthesis. This may be related, at least in part, to increased milk protein synthesis, as protein supplementation increased litter weight gain, which is a proxy for milk production [10]. Furthermore, up-regulation of spliceosomal RNA in particular, indicates that protein supplementation may actually result in increased rate of post-transcriptional modifications of synthesised proteins [33], [34], [35], which in turn may be involved in immune-regulation. Another consequence of protein supplementation was increased cell turnover which may be beneficial in the case of parasitized hosts, as increased cell turnover has been linked to elimination of gut nematodes. Accelerated cell turnover in the large intestine was shown to be one of the main factors contributing to higher rate of *Trichuris muris* expulsion in mice genetically resistant to infection [36]. This increased rate of cell turnover was shown to be under immune control, orchestrated by IL-13 and CXCL10, which was not evident in our current study. Consequently it seems possible that nutritionally and genetically improved worm expulsion may arise from similar effector mechanisms, which are mediated in a different manner. In support of this hypothesis, our results show that there is an increase in regulation of ribosomal proteins in HP rats compared to LP rats. This has also been reported in sheep resistant to gastrointestinal nematodes compared to their more susceptible counterparts [37]. Such an increase has been associated with accelerated cell growth and differentiation [38]; interestingly this appears to be observed in hosts resistant to nematodes and in those supplemented with protein.

In addition to the up-regulation of genes with a function in accelerated cell turnover in HP rats, other genes with possible role in the regulation of immune responses to nematodes seem to be affected by protein nutrition. *IL1R2* and *VSIG4* were both up-regulated in HP rats, whereas *CD3G* was down-regulated. *IL1R2* encodes a cytokine receptor that binds to pro-inflammatory cytokines IL1A, IL1B and IL1R1 and acts as a decoy receptor that inhibits their activity [39]. *VSIG4* encodes a B7 family member protein, and is expressed in macrophages, dendritic cells and neutrophils. It is involved in the negative regulation of the pro-inflammatory cytokine IL-2 and consequently decreases proliferation of T-lymphocytes [40]. Furthermore, vanin 1 (*VNN1*) was down-regulated in the intestine of HP rats, and it is known for its pro-inflammatory role. VNN1 antagonises the activity of peroxisome proliferator activated receptor gamma and is a mediator of inflammation by epithelial cells [41]. These findings imply that at the specific sampling point, LP animals showed increased signs of T-cell proliferation and possibly generalised inflammation, compared to HP animals. Increased expression of *CD3G* in LP rats, which is a T-cell receptor, further supports the view of increased number of T-cells in LP animals in the intestinal mucosa. This could be due to delayed responses in the LP animals whereby T cell responses are reaching a peak at day 6 post secondary infection, in comparison to the T cell response in HP animals which may have peaked earlier and are now being down-regulated to allow for immunoregulation and tissue repair. In support of this view, Jones et al [3] observed that HP diets elevated local antibody production as early as day 3 post infection, suggesting that protein supplementation may at that time induce mechanisms leading to worm expulsion observed a few days later. It is also plausible that an LP diet may result in disordered inflammation and damage in the gastrointestinal tract, whereas HP diet may result in a controlled, protective immune response followed by increased regulation of immunity, which is effective towards parasite expulsion and tissue repair respectively. Furthermore, protein deficiency has been associated with altered T-cell subsets [42]; thus we can not exclude the possibility that although LP hosts had higher numbers of T-cells here, these cells may have altered, and perhaps inappropriate T-cell activity.

Our findings are also in support of the observations made by Tu et al [43] who reported up-regulation of pro-inflammatory cytokines, led by IFN-γ, in protein deficient mice infected with *Heligmosomoides bakeri* during a primary infection. Although under conditions of nutrient adequacy *H. bakeri* induces a strong Th2 response which facilitates parasite expulsion, it appears that when protein nutrition is deficient this cytokine profile is altered. Instead, a Th1 cytokine response is predominant, which is known to promote *H. bakeri* survival [44]. It has been hypothesised that this change in the cytokine pattern is mediated via leptin, which is released from the adipocytes following protein deprivation, which results in up-regulation of IFN-γ and a Th1 response [43]. In agreement, the network analysis on the effects of protein supplementation on gene expression in our study revealed that there is a strong link between the genes differentially regulated following protein supplementation and three pro-inflammatory cytokines: IL1B, IL2, TNF (Figure 2). Neither of the above two routes can be excluded, i.e. whether the increased signs of inflammation in LP rats are the outcome of delayed immune responses or a pro-inflammatory response mediated by protein deprivation. Further investigation on the temporal effects of protein deprivation on pro- and anti-inflammatory immune responses to nematodes is required to shed light into the underlying mechanisms of this interesting outcome.

Despite significantly lower worm burdens in HP rats compared to the LP rats [6], [10], the analysis of the interaction between the level of the infection and the dietary protein showed that under the conditions tested in this study this difference was not significant at the transcriptional level. From the genes selected for validation, *FOXP1* tended to be higher in parasitized rats offered the LP diet compared to the other infection-diet combinations, whereas expression data for two other candidate genes (*GYG1* and *AGR2*) were not confirmed. *FOXP1* is a transcription regulator commonly expressed in B-cells [45] and is involved in early T-cell development, macrophage differentiation and B-cells production [46]. This tendency for *FOXP1* to be up-regulated in LP rats may be related to delayed immune responses in LP rats as discussed above. The earlier referred to observation that HP diets result in early elevated local antibody production [3] could be seen as in support of this view. The gene may have an early effect on infection process, possibly related to the early presence of antibody-producing B cells in the intestinal tissue. Indeed, it appears that despite down-regulation of *FOXP1* in HP rats, *FCGR3A* was up-regulated. This was in agreement with the increased, marginally failing to reach significance, circulating IgG in HP rats [6].

There are three possible reasons for the absence of significant effects on the interactions between dietary protein and the parasitism: (i) lack of power to investigate the interaction in the current study (n = 6), although the sample size was deemed appropriate to study worm kinetics in the rat small intestine [6], [10] (ii) the differences at the transcript level occurred earlier in the experimental study or (iii) there were no differences in the interaction at the transcript level. It is also possible that the beneficial effects of protein supplementation on the host are evident irrespective of the pathogen challenge of the host, i.e. the effects of protein on gene regulation may be evident in the absence or presence of a parasite challenge. This appears to be the case in sheep genetically susceptible to parasitic infection, as they seem to generate a hyper-sensitive immune response, with a number of genes related to stress and infection highly expressed even in the absence of nematodes [37], [47]. Furthermore, the consistently observed phenotype in all studies with this infection model (low worm burden in HP rats) may be related, at least partly, to the increased cell metabolism and turn over of the epithelial cells. However, previously established differences between mast cell and goblet cell numbers and mucosal antibodies [3], [6], [19] denote that there should also be differences in gene regulation that were not identified in the present study.

By using a genome-wide transcriptomic analysis this study provides significant insights into the immune-modulatory effects of nutrition. We demonstrate that the nutritional environment of the host can affect gene regulation at transcript level during the phase of parasite expulsion in parasitized, but also in primed, non-parasitized hosts. We provided evidence that HP environment resulted in increased cell turnover which may have a role in parasite expulsion and that at the time of sampling, HP diet may result in increased immune-regulation, but reduced inflammation of the intestinal tissue compared to a LP diet. Our data also demonstrated a link between protein supplementation and immunity; the top three canonical pathways significantly affected by the HP environment have a role in immune-modulation, i.e. sphingolipid metabolism [48], PPAR signalling [49] and p38 MAPK signalling [50]. Genes associated with innate immunity and oxidative metabolism, such as *AGR2*, *DUOX2* and *DUOXA2* were shown to be up-regulated during the secondary challenge, implying a possible role in parasite expulsion. Experiments on the temporal effects of the genes identified in this study and their role in nematode expulsion under different nutritional regimes will advance our knowledge and enable us to map the interactions between dietary protein and parasitism. The work presented here is the first important step towards this goal.

## Methods

### Experimental animals and treatments

The animal experiment was approved by SAC's Ethical Review Committee (ED AE 24/2007) and carried out under Home Office authorization (PPL 60/3626).Twenty-three second parity female Sprague Dawley rats (Charles River UK Ltd, Kent, UK) were housed individually. Fourteen days prior to mating, all rats were infected subcutaneously in the hind limb with 1600 third-stage infective larvae of *N. brasiliensis* in 0.5 ml sterile PBS according to a previously established protocol [10]. A secondary infection with 1600 third-stage infective larvae (P; n = 12) was administered on day 2 of lactation (in average 24 days post conception). At the same time point, control uninfected rats received a sham infection with 0.5 ml sterile PBS (NP; n = 11; one of the rats was put down for ethical reasons not related to the experimental design).

From the primary infection and until mating was confirmed, rats were given *ad libitum* access to standard rat chow (B&K Universal Ltd, Hull, UK). Mated rats were then given *ad libitum* access to a food containing 210 g digestible crude protein (CP) and 16.4 MJ metabolisable energy (ME) per kg dry matter (DM) for the first 10 days of gestation followed by a low protein food containing 60 g CP and 17.3 MJ ME per kg DM until parturition. This feeding protocol has been used to reduce body protein reserves during the second half of gestation to maximise the degree of protein scarcity when rats are fed low protein foods during lactation and induce PPRI [51], [52].

From parturition onwards, rats were given a restricted amount of either a low protein (LP) food, with 130 g CP per kg DM or a high protein (HP) food, with 270 g CP per kg DM. The feeds were kept iso-energetic at 14 MJ ME per kg DM by exchanging casein for starch to achieve the different CP contents; the ratio of individual amino acids to CP was constant between the two foods. The restricted amount of food offered to rats was calculated to be 90% of achieved ME intake on similar low and high protein diets [3]. The restricted allowance of the experimental diets was based on dam parturition weight. Weight of food offered increased over time to account for the increasing milk demand from growing pups. Rats were euthanized on day 6 post secondary infection, as previous and present animal studies on the same infection model have demonstrated that the differences in parasite burdens between LP and HP fed animals was the greatest at this time point [3], [6], [10]. Duodenal samples were fixed in RNA later (Sigma, UK) and were stored at −80°C until RNA extraction.

### RNA extraction and cDNA synthesis

Total RNA was isolated by adding 1 ml of Trizol (Invitrogen, Paisley, UK) to the gut samples and homogenising the tissue with ceramic beads (CK28 tubes; Stretton Scientific, Stretton, UK) using a Precellys tissue homogeniser (Bertin Technologies, Montigny-le-Bretonneux, France). Total RNA was then isolated following a protocol based upon the single-step acid guanidiunium thiocyanate-phenol-chloroform RNA isolation method [53]. Following the extraction, RNA yield was determined by NanoDrop ND-1000 spectrophotometer at 260/280 nm (Nanodrop Technologies Inc). Quality and integrity of total RNA were further analysed with an Agilent Bioanalyser (Agilent Technologies, UK). The RNA integrity number for each sample (RIN) was obtained; all of the RNA samples used for the arrays had RINs higher than 7.5 and were therefore deemed to be of acceptable quality [54]. The cDNA for quantitative PCR was synthesised using verso cDNA synthesis kit following manufacturer's instructions (1 µg RNA per reaction; Thermo Scientific, UK). The kit encompasses a genomic DNA eliminator column, and achieves elimination from genomic DNA and cDNA synthesis in one step. Once cDNA was synthesised it was stored at −20°C until use.

### Microarray experimental design and analysis

The Affymetrix Rat Gene 1.0 ST array was used for gene expression analysis (Affymetrix UK Ltd). The array scans the whole genome with over 700,000 probes constituting over 27,000 gene level probe sets. Hybridization and labelling of the array were performed following standard operating protocols at ARK Genomics (Roslin, UK). After hybridization, the arrays were washed on the Genechip Fluidics Station 450 and scanned with the Genechip Scanner 3000 according to the manufacturer's protocols (Affymetrix UK Ltd). Raw microarray data were processed and analyzed within GeneSpring (GX 11.0, Agilent, UK). Numerical quality control steps were applied to each of the 23 samples. Arrays were normalized and converted to probe-level data with Robust Multi-Array Average (RMA) [55]. The experiment was analysed as a 2×2 factorial design, with two levels of protein (LP and HP) and two levels of infection (P and NP). We have deposited the raw data at ArrayExpress under accession number E-TABM-1132; we can confirm all details are MIAME compliant as detailed on the MGED Society website http://www.mged.org/Workgroups/MIAME/miame.html.

The statistical analysis was performed within Genespring GX 11.0 and in order to limit multiple testing errors, a filter on probe expression values was applied to reduce the number of probes used in the statistical analysis. This filter was designed to remove genes that were relatively lowly expressed in all samples. Prior to the application of the filter 27,342 probes were present and the filtering reduced this to 16,510 probes for the differential expression analysis. A two-way ANOVA was then performed at a significance level of P≤0.05 and results were further corrected for multiple testing errors (MTC) using the false discovery rate (FDR) algorithm developed by Benjamini-Hochberg [56], at cut-off of P≤0.05. Following the application of the FDR correction no significantly differentially expressed genes were identified for the interaction between dietary and infection treatment. For this reason the FDR correction was not applied for the interaction data, and the results were interpreted with the caveat that all of the genes could represent false positives. Finally, to generate the final lists of candidate genes that changed in response to the various treatments, differential expression (by at least 1.5-fold for the main effects and 1.1 fold for the interaction) was also applied. Clustering analysis was performed within Genespring GX 11.0, where clusters of genes with similar expression patterns were identified and then separately analysed using Ingenuity Pathways Analysis (IPA) software v8.5 (Ingenuity Systems, CA). Transcriptional promoter analysis was performed with the oPOSSUM software, using the mouse database and the default settings [57].

Gene function, network and pathway analysis were performed using IPA. Each gene identifier was mapped to its corresponding gene object in the Ingenuity Pathways Knowledge Base. Gene networks were then algorithmically generated based on their connectivity and assigned a score. The score is a numerical value used to rank networks according to how relevant they are to the genes in the input dataset. Analysis of transcription networks also enabled the identification of additional co-regulated genes that may have been otherwise missed. IPA uses a right-tailed Fisher's test to calculate the p-value for networks. A score of 10 indicates a p = 10^−10^ chance that genes in that network are associated solely by chance. The networks created by the software consisted of nodes (representing the genes) connected by edges (representing the connections between genes based on biological relationships identified from the scientific literature). The intensity of the node colour indicates the degree of up- (red) or down- (green) regulation. Nodes are displayed using various shapes that represent the functional class of the gene product, and edges are displayed with various labels indicating the nature of the relationship between the nodes as described in the figure legends. Canonical pathway analysis identified the biological pathways of most significance to the input data sets. The significance of the association between the data set and the canonical pathway was determined based on two parameters: (1) A ratio of the number of genes from the data set that map to the pathway divided by the total number of genes that map to the canonical pathway and (2) a p-value calculated using Fisher's exact test determining the probability that the association between the genes in the data set and the canonical pathway is due to chance alone.

### Real-Time Quantitative Reverse Transcriptase Polymerase Chain Reaction

Quantitative PCR was performed on an ABI Prism 7000® QPCR thermocyler (Applied Biosystems, Foster City, USA). cDNA was diluted 10-fold and 1 µl added to a 20 µl reaction, also containing 10 µl 2× JumpStart Taq ReadyMix (16 µM Tris-HCl, pH 8,3, 80 µM KCl, 5.6 MgCl2, 320 nM dNTP, stabilizers, 0.5 U Taq DNA polymerase, JumpStart Taq antibody, and SYBR Green I; Sigma). A range of candidate genes were identified as targets for qPCR (Table 9). Primers (Table 9) were either previously published or designed with the online Primer Blast software (http://www.ncbi.nlm.nih.gov/tools/primer-blast/) and tested in Net Primer Software (http://www.premierbiosoft.com/netprimer/index.html). All primers were purchased from Invitrogen (Paisley, UK). The thermal profile of the reactions included: 2 min at 50°C, 10 min at 95°C, then 40 cycles at 95°C for 30 seconds, annealing temperatures for 1 minute and 72°C for 30 seconds. Real time PCR was performed with melting curve analysis to establish the purity of each amplified product. Absolute mRNA levels were calculated for genes using an included standard curve for each individual gene and values normalised to the housekeeping gene β-actin. Gene portions were cloned into pDRIVE (Qiagen, UK) to create standard curves.

**Table 9:** Primer sequence, annealing temperature (AT°C) and PCR product length for genes amplified using qPCR.

Gene name	Forward Primer	Reverse Primer	AT°C	PCR product (bp)
*β-actin*	CGTTGACATCCGTAAAGACC	TAGAGCCACCAATCCACAC	60	176
*AGR2*	CAAAGGACTCTCGACCCAAA	ACTGCTCTGCCAATTTCTGG	61.5	213
*IL4*	ACCTTGCTGTCACCCTGTTCTGC	GTTGTGAGCGTGGACTCATTCACG	60	352
*IL13*	CTCAGGGAGCTTATCGAGGA	CGAGGCCTTTTGGTTACAGA	60	201
*G6PC*	TGGCCTGGCCTACTGCACCT	GTTCCCGCTCCATGGGCACC	59.5	149
*ARHGAP15*	CAGATTGCCAAAGAATCCAAGTTTCGG	TCAGTCTGCTCTTCACGCGGT	59.5	194
*ADH1C*	AGGGAGCTTTGCTCGACGGC	GCGACTTGGACGGCAGAGCC	59.5	202
*GYG1*	CCAGACCCAGGGTGGCCTGA	ACCAAACGCTTTAAATGCCGGGAG	61.5	246
*FOXP1*	ACGTGCCCATTTCTTCAGCAGAT	CGTGGCTGCATTGCGTCGGA	61.5	194
*RETNLB*	GCCCATCGAGATAACTGTCCCTCC	CGGGCAGTGGCCCAATCCAT	61.5	200
*CD3G*	CTGGGCAACGGTGCCAAAGA	GGCGAACTCCATCCTGTCCAGC	61.5	211
*IL1R2*	ACATCCATGGGAGATGCAGGCT	CCAGGAACACCTTGCACGGGA	61.5	175

Statistical significance was determined by a two-way ANOVA, with protein and parasite challenge as factors (Genstat 11^th^ edition). For all comparisons, P<0.05 was considered significant, whereas observed tendencies (0.05<P<0.1) are also reported accordingly.

## Supporting Information

Table S1 **Extended list of significantly differentially regulated by secondary challenge with** ***N.brasiliensis*** **in lactating rats (P<0.05, FC>1.5, MTC 0.05).**
(DOCX)

Table S2 **Genes differentially expressed as a consequence of the interaction between protein supplementation and a secondary** ***N.brasiliensis*** **challenge in lactating rats (P<0.05, FC>1, No MTC).**
(DOCX)
