# Supplementary material for: Genome-Wide Transcriptomic Analysis of Intestinal Tissue to Assess the Impact of Nutrition and a Secondary Nematode Challenge in Lactating Rats
Source: PLoS One. 2011 Jun 16;6(6):e20771. doi: 10.1371/journal.pone.0020771 (PMC3116830; doi:10.1371/journal.pone.0020771)
Supplement: Table S1 — Extended list of significantly differentially regulated by secondary challenge with N.brasiliensis in lactating rats (P<0.05, FC>1.5, MTC 0.05). (DOCX) [file pone.0020771.s001.docx]

Table S1. Extended list of significantly differentially regulated by secondary challenge with *N.brasiliensis* in lactating rats (P<0.05, FC>1.5, MTC 0.05).

| Gene symbol | Gene description | Fold Change | Location | Type(s) |
| --- | --- | --- | --- | --- |
| *DUOXA2* | Dual oxidase maturation factor 2 | 11.8 | Cytoplasm | other |
| *DUOX2* | Dual oxidase 2 | 8.3 | unknown | enzyme |
| *MCPT4* | Mast cell protease 4 | 7.7 | Extracellular Space | peptidase |
| *PLA2G4C* | Phospholipase A2, group IVC (cytosolic, calcium-independent) | 4.3 | Cytoplasm | enzyme |
| *RETNLB* | Resistin like beta | 3.9 | Extracellular Space | other |
| *CD55* | CD55 molecule | 3.7 | Plasma Membrane | other |
| *TPSB2* | Tryptase beta 2 | 3.7 | Extracellular Space | peptidase |
| *MCPT8* | Mast cell protease 8 | 3.5 | Extracellular Space | peptidase |
| *TPSG1* | Tryptase gamma 1 | 3.2 | Extracellular Space | peptidase |
| *IL1RL1* | Interleukin 1 receptor-like 1 | 3.1 | Plasma Membrane | transmembrane receptor |
| *GSDMC* | Gasdermin C | 3.0 | Cytoplasm | other |
| *POSTN* | Periostin, osteoblast specific factor | 3.0 | Extracellular Space | other |
| *MCPT1* | Mast cell protease 1 | 2.9 | Extracellular Space | peptidase |
| *CPA3* | Carboxypeptidase A3 (mast cell) | 2.7 | Extracellular Space | peptidase |
| *MCPT4* | Mast cell protease 4 | 2.6 | Extracellular Space | peptidase |
| *GPX2* | Glutathione peroxidase 2 (gastrointestinal) | 2.6 | Cytoplasm | enzyme |
| *CNN1* | Calponin 1, basic, smooth muscle | 2.6 | Cytoplasm | other |
| *SRGN* | Serglycin | 2.5 | Extracellular Space | other |
| *DES* | Desmin | 2.5 | Cytoplasm | other |
| *SPINK4* | Serine peptidase inhibitor, Kazal type 4 | 2.5 | Extracellular Space | other |
| *RGS13* | Regulator of G-protein signaling 13 | 2.5 | Nucleus | other |
| *APOL3* | Apolipoprotein L, 3 | 2.4 | Cytoplasm | transporter |
| *ACTG2* | Actin, gamma 2, smooth muscle, enteric | 2.4 | Cytoplasm | other |
| *ITLN1* | Intelectin 1 (galactofuranose binding) | 2.3 | Plasma Membrane | other |
| *ITGA5* | Integrin, alpha 5 (fibronectin receptor, alpha polypeptide) | 2.2 | Plasma Membrane | other |
| *SH2D6* | SH2 domain containing 6 | 2.2 | unknown | other |
| *LGALS1* | Lectin, galactoside-binding, soluble, 1 | 2.2 | Extracellular Space | other |
| *CMA1* | Chymase 1, mast cell | 2.1 | Extracellular Space | peptidase |
| *MMP10* | Matrix metallopeptidase 10 (stromelysin 2) | 2.0 | Extracellular Space | peptidase |
| *NKG7* | Natural killer cell group 7 sequence | 2.0 | Plasma Membrane | other |
| *TAGLN* | Transgelin | 1.9 | Cytoplasm | other |
| *CBR1* | Carbonyl reductase 1 | 1.9 | Cytoplasm | enzyme |
| *NMU* | Neuromedin U | 1.9 | Extracellular Space | other |
| *SH2D7* | SH2 domain containing 7 | 1.9 | unknown | other |
| *FAM3D* | Family with sequence similarity 3, member D | 1.9 | Extracellular Space | cytokine |
| *FLNC* | Filamin C, gamma | 1.8 | Cytoplasm | other |
| *PLAU* | Plasminogen activator, urokinase | 1.8 | Extracellular Space | peptidase |
| *ALOX5AP* | Arachidonate 5-lipoxygenase-activating protein | 1.8 | Plasma Membrane | other |
| *FHL1* | Four and a half LIM domains 1 | 1.8 | Cytoplasm | other |
| *SLC38A8* | Solute carrier family 38, member 8 | 1.8 | unknown | other |
| *FCGR2B* | Fc fragment of IgG, low affinity IIb, receptor (CD32) | 1.8 | Plasma Membrane | transmembrane receptor |
| *ALOX5* | Arachidonate 5-lipoxygenase | 1.8 | Cytoplasm | enzyme |
| *CFL2* | Cofilin 2 (muscle) | 1.7 | Nucleus | other |
| *FAM151A* | Family with sequence similarity 151, member A | 1.7 | unknown | other |
| *PLTP* | Phospholipid transfer protein | 1.7 | Extracellular Space | other |
| *GZMB* | Granzyme B (granzyme 2, cytotoxic T-lymphocyte-associated serine esterase 1) | 1.7 | Cytoplasm | peptidase |
| *CHI3L1* | Chitinase 3-like 1 (cartilage glycoprotein-39) | 1.7 | Extracellular Space | enzyme |
| *FAM40B* | Family with sequence similarity 40, member B | 1.7 | unknown | other |
| *CCL11* | Chemokine (C-C motif) ligand 11 | 1.7 | Extracellular Space | cytokine |
| *CACNA2D1* | Calcium channel, voltage-dependent, alpha 2/delta subunit 1 | 1.7 | Plasma Membrane | ion channel |
| *TIMP1* | TIMP metallopeptidase inhibitor 1 | 1.7 | Extracellular Space | other |
| *CALD1* | Caldesmon 1 | 1.6 | Cytoplasm | other |
| *CRYAB* | Crystallin, alpha B | 1.6 | Nucleus | other |
| *MS4A2* | Membrane-spanning 4-domains, subfamily A, member 2 | 1.6 | Plasma Membrane | transmembrane receptor |
| *CYSLTR1* | Cysteinyl leukotriene receptor 1 | 1.6 | Plasma Membrane | G-protein coupled receptor |
| *CSRP1* | Cysteine and glycine-rich protein 1 | 1.6 | Nucleus | other |
| *BMX* | BMX non-receptor tyrosine kinase | 1.6 | Cytoplasm | kinase |
| *CLCA1* | Chloride channel accessory 1 | 1.6 | Plasma Membrane | ion channel |
| *FN1* | Fibronectin 1 | 1.6 | Plasma Membrane | enzyme |
| *PGM5* | Phosphoglucomutase 5 | 1.6 | Cytoplasm | enzyme |
| *NRG1* | Neuregulin 1 | 1.6 | Extracellular Space | growth factor |
| *nPMM1* | Phosphomannomutase 1 | 1.6 | Cytoplasm | enzyme |
| *BCHE* | Butyrylcholinesterase | 1.6 | Plasma Membrane | enzyme |
| *SERPINE1* | Serpin peptidase inhibitor, clade E, member 1 | 1.6 | Extracellular Space | other |
| *TUBB6* | Tubulin, beta 6 | 1.6 | Cytoplasm | other |
| *MYL9* | Myosin, light chain 9, regulatory | 1.5 | Cytoplasm | other |
| *DCLK1* | Doublecortin-like kinase 1 | 1.5 | Cytoplasm | kinase |
| *HSPB1* | Heat shock 27kDa protein 1 | 1.5 | Cytoplasm | other |
| *MCTP2* | Multiple C2 domains, transmembrane 2 | 1.5 | unknown | other |
| *SLC2A1* | Solute carrier family 2 (facilitated glucose transporter), member 1 | 1.5 | Plasma Membrane | transporter |
| *COL6A3* | Collagen, type VI, alpha 3 | 1.5 | Extracellular Space | other |
| *AGR2* | Anterior gradient homolog 2 (Xenopus laevis) | 1.5 | Extracellular Space | other |
| *CD44* | CD44 molecule (Indian blood group) | 1.5 | Plasma Membrane | other |
| *PTGS2* | Prostaglandin-endoperoxide synthase 2 (prostaglandin G/H synthase and cyclooxygenase) | 1.5 | Cytoplasm | enzyme |
| *PLA2G7* | Phospholipase A2, group VII (platelet-activating factor acetylhydrolase, plasma) | 1.5 | Extracellular Space | enzyme |
| *ACTN1* | Actinin, alpha 1 | 1.5 | Cytoplasm | other |
| *PCP4* | Purkinje cell protein 4 | 1.5 | Cytoplasm | other |
| *CXCL11* | Chemokine (C-X-C motif) ligand 11 | -1.6 | Extracellular Space | cytokine |
| *PBLD* | Phenazine biosynthesis-like protein domain containing | -1.6 | unknown | enzyme |
| *SCARB1* | Scavenger receptor class B, member 1 | -1.6 | Plasma Membrane | transporter |
| *MAOB* | Monoamine oxidase B | -1.7 | Cytoplasm | enzyme |
| *ALDH8A1* | Aldehyde dehydrogenase 8 family, member A1 | -1.7 | unknown | other |
| *DNASE1* | Deoxyribonuclease I | -2.0 | Extracellular Space | enzyme |
| *G6PC* | Glucose-6-phosphatase, catalytic subunit | -3.2 | Cytoplasm | phosphatase |
