# Supplementary material for: Genome-Wide Transcriptomic Analysis of Intestinal Tissue to Assess the Impact of Nutrition and a Secondary Nematode Challenge in Lactating Rats
Source: PLoS One. 2011 Jun 16;6(6):e20771. doi: 10.1371/journal.pone.0020771 (PMC3116830; doi:10.1371/journal.pone.0020771)
Supplement: Table S2 — Genes differentially expressed as a consequence of the interaction between protein supplementation and a secondary N.brasiliensis challenge in lactating rats (P<0.05, FC>1, No MTC). (DOCX) [file pone.0020771.s002.docx]

Table S2. Genes differentially expressed as a consequence of the interaction between protein supplementation and secondary challenge (P<0.05, FC>1, No MTC)

| Gene symbol | Gene description | Location | Type(s) |
| --- | --- | --- | --- |
| *ACYP2* | Acylphosphatase 2, muscle type | unknown | enzyme |
| *ADRA1A* | Adrenergic, alpha-1A-, receptor | Plasma Membrane | G-protein coupled receptor |
| *ADRBK2* | Adrenergic, beta, receptor kinase 2 | Cytoplasm | kinase |
| *AGR2* | Anterior gradient homolog 2 (Xenopus laevis) | Extracellular Space | other |
| *AGTPBP1* | ATP/GTP binding protein 1 | Nucleus | peptidase |
| *ANKS1B* | Ankyrin repeat and sterile alpha motif domain containing 1B | Nucleus | other |
| *ARHGAP15* | Rho GTPase activating protein 15 | unknown | other |
| *ARIH2* | Ariadne homolog 2 (Drosophila) | Nucleus | other |
| *B4GALT6* | UDP-Gal:betaGlcNAc beta 1,4- galactosyltransferase, polypeptide 6 | Cytoplasm | enzyme |
| *CALCRL* | Calcitonin receptor-like | Plasma Membrane | G-protein coupled receptor |
| *CENPA* | Centromere protein A | Nucleus | other |
| *CENPI* | Centromere protein I | Nucleus | other |
| *CIB3* | Calcium and integrin binding family member 3 | unknown | other |
| *COL7A1* | Collagen, type VII, alpha 1 | Extracellular Space | other |
| *CPD* | Carboxypeptidase D | Extracellular Space | peptidase |
| *CSMD2* | CUB and Sushi multiple domains 2 | unknown | other |
| *CUL1* | Cullin 1 | Nucleus | enzyme |
| *DCUN1D3* | DCN1, defective in cullin neddylation 1, domain containing 3 (S. cerevisiae) | unknown | other |
| *DKK4* | Cickkopf homolog 4 (Xenopus laevis) | Extracellular Space | other |
| *ELF1* | E74-like factor 1 (ets domain transcription factor) | Nucleus | transcription regulator |
| *FCGR3A* | Fc fragment of IgG, low affinity IIIa, receptor (CD16a) | Plasma Membrane | transmembrane receptor |
| *FOXF2* | Forkhead box F2 | Nucleus | transcription regulator |
| *FOXL2* | Forkhead box L2 | Nucleus | transcription regulator |
| *FOXP1* | Forkhead box P1 | Nucleus | transcription regulator |
| *GPD2* | Glycerol-3-phosphate dehydrogenase 2 (mitochondrial) | Cytoplasm | enzyme |
| *GPR12* | G protein-coupled receptor 12 | Plasma Membrane | G-protein coupled receptor |
| *GSC* | Goosecoid homeobox | Nucleus | transcription regulator |
| *GYG1* | Glycogenin 1 | Cytoplasm | enzyme |
| *HEY1* | Hairy/enhancer-of-split related with YRPW motif 1 | Nucleus | transcription regulator |
| *HIVEP1* | Human immunodeficiency virus type I enhancer binding protein 1 | Nucleus | transcription regulator |
| *ITFG1* | Integrin alpha FG-GAP repeat containing 1 | Plasma Membrane | other |
| *KCNJ4* | Potassium inwardly-rectifying channel, subfamily J, member 4 | Plasma Membrane | ion channel |
| *KIF5B* | Kinesin family member 5B | Cytoplasm | other |
| *KRTAP10-8* | Keratin associated protein 10-8 | unknown | other |
| *LSM6* | LSM6 homolog, U6 small nuclear RNA associated (S. cerevisiae) | Nucleus | other |
| *MASTL* | Microtubule associated serine/threonine kinase-like | unknown | kinase |
| *MPP4* | Membrane protein, palmitoylated 4 (MAGUK p55 subfamily member 4) | Cytoplasm | kinase |
| *MYH7B (includes EG:57644)* | Myosin, heavy chain 7B, cardiac muscle, beta | unknown | other |
| *NKX2-4* | NK2 homeobox 4 | Nucleus | transcription regulator |
| *OLR1* | Oxidized low density lipoprotein (lectin-like) receptor 1 | Plasma Membrane | transmembrane receptor |
| *OXT* | Oxytocin, prepropeptide | Extracellular Space | other |
| *PAFAH1B2* | Platelet-activating factor acetylhydrolase, isoform Ib, subunit 2 (30kDa) | Cytoplasm | enzyme |
| *PLEKHA3* | Pleckstrin homology domain containing, family A (phosphoinositide binding specific) member 3 | Cytoplasm | other |
| *PPP1R3C* | Protein phosphatase 1, regulatory (inhibitor) subunit 3C | Cytoplasm | phosphatase |
| *PPP2R2D* | Protein phosphatase 2, regulatory subunit B, delta isoform | Nucleus | phosphatase |
| *PRKX* | Protein kinase, X-linked | Cytoplasm | kinase |
| *PTPRU* | Protein tyrosine phosphatase, receptor type, U | Plasma Membrane | phosphatase |
| *RAB10* | RAB10, member RAS oncogene family | Cytoplasm | enzyme |
| *RADIL* | Ras association and DIL domains | unknown | other |
| *RAP1GDS1* | RAP1, GTP-GDP dissociation stimulator 1 | unknown | other |
| *RC3H2* | Ring finger and CCCH-type zinc finger domains 2 | Plasma Membrane | other |
| *RIOK1* | RIO kinase 1 (yeast) | unknown | kinase |
| *RNF2* | Ring finger protein 2 | Nucleus | transcription regulator |
| *SAV1* | Salvador homolog 1 (Drosophila) | unknown | other |
| *SLC7A5* | Solute carrier family 7 (cationic amino acid transporter, y+ system), member 5 | Plasma Membrane | transporter |
| *SNRNP35* | Small nuclear ribonucleoprotein 35kDa (U11/U12) | unknown | other |
| *ST7* | Suppression of tumorigenicity 7 | unknown | other |
| *TLE2* | Transducin-like enhancer of split 2 (E(sp1) homolog, Drosophila) | Nucleus | transcription regulator |
| *TMEM158* | Transmembrane protein 158 | Plasma Membrane | other |
| *UBA52* | Ubiquitin A-52 residue ribosomal protein fusion product 1 | Cytoplasm | transcription regulator |
| *UBB* | Ubiquitin B | Cytoplasm | other |
| *UPRT* | Uracil phosphoribosyltransferase (FUR1) homolog (S. cerevisiae) | unknown | other |
| *USP25* | Ubiquitin specific peptidase 25 | unknown | peptidase |
| *WRB* | Tryptophan rich basic protein | Extracellular Space | other |
| *ZG16* | Zymogen granule protein 16 homolog (rat) | Extracellular Space | other |
| *ZNF354A* | Zinc finger protein 354A | Nucleus | transcription regulator |
